# Genetic risk assessment based on association and prediction studies

**DOI:** 10.1038/s41598-023-41862-3

**Published:** 2023-09-14

**Authors:** Nicole Cathlene N. Astrologo, Joverlyn D. Gaudillo, Jason R. Albia, Ranzivelle Marianne L. Roxas-Villanueva

**Affiliations:** 1 Data Analytics Research Laboratory (DARELab), Institute of Mathematical Sciences and Physics, University of the Philippines Los Baños, 4031 Los Baños, Laguna Philippines; 2 Computational Interdisciplinary Research Laboratory (CINTERLabs), University of the Philippines Los Baños, 4031 Los Baños, Laguna Philippines; 3 Domingo AI Research Center (DARC Labs), 1606 Pasig, Philippines; 4 Venn Biosciences Corporation Dba InterVenn Biosciences, Metro Manila, Pasig, Philippines; 5 Graduate School, University of the Philippines Los Baños, 4031 Los Baños, Laguna Philippines

**Keywords:** Genetics, Genomic instability, Predictive markers

## Abstract

The genetic basis of phenotypic emergence provides valuable information for assessing individual risk. While association studies have been pivotal in identifying genetic risk factors within a population, complementing it with insights derived from predictions studies that assess individual-level risk offers a more comprehensive approach to understanding phenotypic expression. In this study, we established personalized risk assessment models using single-nucleotide polymorphism (SNP) data from 200 Korean patients, of which 100 experienced hepatitis B surface antigen (HBsAg) seroclearance and 100 patients demonstrated high levels of HBsAg. The risk assessment models determined the predictive power of the following: (1) genome-wide association study (GWAS)-identified candidate biomarkers considered significant in a reference study and (2) machine learning (ML)-identified candidate biomarkers with the highest feature importance scores obtained by using random forest (RF). While utilizing all features yielded 64% model accuracy, using relevant biomarkers achieved higher model accuracies: 82% for 52 GWAS-identified candidate biomarkers, 71% for three GWAS-identified biomarkers, and 80% for 150 ML-identified candidate biomarkers. Findings highlight that the joint contributions of relevant biomarkers significantly influence phenotypic emergence. On the other hand, combining ML-identified candidate biomarkers into the pool of GWAS-identified candidate biomarkers resulted in the improved predictive accuracy of 90%, demonstrating the capability of ML as an auxiliary analysis to GWAS. Furthermore, some of the ML-identified candidate biomarkers were found to be linked with hepatocellular carcinoma (HCC), reinforcing previous claims that HCC can still occur despite the absence of HBsAg.

## Introduction

Since the genetic architecture of complex diseases follows a polygenic rather than a Mendelian model^1–4^, understanding disease emergence and progression through gaining insights into genomic instability continues to challenge researchers. While genomic instability reveals only a portion of the biological underpinnings of complex diseases^5–9^, identifying genetic biomarkers can facilitate targeted and personalized treatments for individuals with increased genetic susceptibility to specific diseases.

Genome-wide association studies (GWAS) serve as the gold standard approach in identifying disease susceptibility variants, such as single nucleotide polymorphisms (SNPs)^10^, associated with complex traits. The study design of GWAS involves testing individual SNPs for their association with the phenotype^11–15^. To come up with a statistically relevant association amidst multiple SNP testing, highly conservative thresholding is necessary, often leading to underpowered SNP detection with small effect sizes^16–19^. Most identified associations point to larger regions of correlated variants due to linkage disequilibrium^20–22^, highlighting the potential influence of neighboring variants with modest effects on predicting phenotypic expression. Similarly, while biomarkers having robust associations are often perceived as prime candidates for modeling, they might be poor predictors of phenotypic outcomes^23^. Assessing the predictive utility of GWAS-identified candidate biomarkers, therefore, still warrants further investigation.

Traditionally, predictive models such as polygenic risk score models were used to quantify the predictive value of SNPs; however, such models are limited to learning only the linear interactions among variables^24–27^. In addition, the “curse of dimensionality,” resulting from the millions of features present in genomic data^28^, prevents attaining an optimized model performance due to the presence of irrelevant features. In the context of personalized medicine, understanding the differences in goals of association and prediction studies^23^ and accounting for the complex interactions among SNPs is a crucial consideration. Machine learning (ML) is a widely accepted methodical framework in analyzing high-dimensional and complex data^6,8,29–32^, owing to its unparalleled ability to handle high-volume data and uncover implicit and nonlinear patterns that are pertinent for predictive modeling. By selecting a minimum subset of individually relevant and neighboring features while minimizing information loss^33^, ML captures complex interactions, leading to the identification of highly-predictive features.

Understanding the differences in the information gained from population and individual levels^23^, this paper aims to identify biomarkers linked to the phenotype by incorporating insights from both analyses. More precisely, this study intends to develop a robust risk assessment model that identifies the best combination of GWAS-identified and ML-identified candidate biomarkers. We organized the study as follows: Section II outlines the data description and preprocessing, as well as the model framework, including feature selection, model classification, and model evaluation through hyperparameter tuning and cross-validation; Section III presents the results of implementing the model framework to various biomarker types such as GWAS-identified candidate biomarkers and ML-identified candidate biomarkers; Section IV discusses the results and key findings; and Section V summarizes main points and their implications in the biomedical field, demonstrates limitations of the study, and provides recommendations for future research.

## Methods

### Data description and preprocessing

The secondary SNP dataset used in this study was obtained from Kim et al.^14^, a study on hepatitis B virus (HBV) surface antigen (HBsAg) seroclearance in patients with chronic hepatitis B (CHB) of homogeneous viral genotype. The data is composed of 200 subjects genotyped for 2,372,784 SNPs. The SNP dataset was subjected to quality control procedures as discussed by Kim et al.^14^: (1) SNPs that were not located on autosomal chromosomes, (2) SNPs with missing call rates of less than 0.95 in both cases and controls, (3) SNPs with a minor allele frequency of less than 0.01, or (4) SNPs with significant deviation from the Hardy-Weinberg equilibrium of $p < 1.0 \times 10^{-5}$ in both case and controls. After the series of exclusion criteria, the total number of SNPs was reduced to 1,318,897.

The phenotypes include 100 patients who underwent HBsAg seroclearance before the age of 60 and 100 patients who demonstrated a high level (> 1000 IU/mL) of HBsAg after the age of 60 as case and control, respectively. HBsAg seroclearance is the absence of circulating HBsAg with or without the presence of antibodies in patients with CHB, hence considering it a functional cure for the infection^14,34^. For further details regarding the data, refer to this paper^14^. Furthermore, the SNPs were encoded in the additive encoding scheme^35^, which counts the number of minor alleles in the phenotype for a suitable representation for ML analysis.

### Model framework

**Figure 1:**
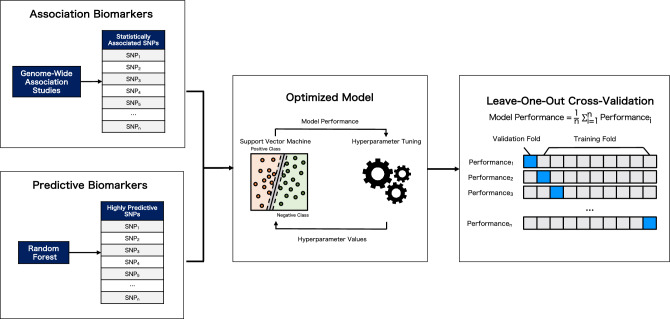
General workflow of the study.

We developed a model framework that determines the best combination of GWAS-identified candidate biomarkers reported in the recent paper^14^ and ML-identified candidate biomarkers through feature selection via random forest (RF). In investigating the most effective approach, the support vector machine (SVM) will be trained using various biomarkers sets, i.e., GWAS-identified candidate biomarkers only, ML-identified candidate biomarkers only, and a combination of both, and assess their classification performances. To maintain consistency in the model configurations, we used identical sets of hyperparameters to optimize SVM. We also implemented a cross-validation scheme during model evaluation to ensure generalizable model performances. Figure 1 illustrates the general workflow adopted in this study.

#### Feature selection

Feature selection is an essential step in ML that reduces the dimensionality of data by selecting the most relevant and informative features to build highly predictive models. Owing to its remarkable ability to build a predictive model without any prior assumptions about the genotype-phenotype relationship^36^, RF was used as a feature selection technique to determine the optimal combination of SNPs. RF uses bootstrapping to train decision trees on randomly sampled subsets of training data, then consolidates the predictions of the individual trees to generate a final prediction. Furthermore, RF enhances the diversity of its ensemble by incorporating randomization at the node level when growing each individual tree by selecting a random feature subset to determine the best split for each node. The feature importance of $SNP_{i}$ is calculated by summing the decrease in Gini impurity $\Delta {I}$ for all nodes *t*. The feature importance of an $SNP_{i}$ is defined in Eq. (1).

1 $$\begin{aligned} FI_{gini}(SNP_{i})=\sum _{t\in T_k}~p(t) ~ \Delta {I} \end{aligned}$$

where $T_{k}$ is the number of nodes in the $k^{th}$ tree, $p(t)= \frac{n_t}{n}$ being the fraction of reaching node *t*, and $\Delta {I}$ being the decrease in Gini impurity.

RF is widely used in “large p, small n” problems due to its one-step-at-a-time node strategy, while still being able to consider correlations and interactions among predictors due to the “grouping property” of decision trees^37^. RF’s inherent ability to capture SNP-SNP interactions and subsequently leading to satisfactory phenotype prediction performance^38–40^, make it suitable for genomic data analysis and bioinformatics research.

#### Hyperparameter tuning

Hyperparameter tuning is necessary for achieving optimal performance in model training. Bayesian Optimization (BayesOpt), a global optimization method for black-box functions such as ML models, was utilized to tune the hyperparameters of RF and SVM. Unlike grid and manual search algorithms wherein experiments are conducted in isolation, BayesOpt balances exploration to uncertain search spaces and exploitation of results from previous experiments to arrive at global rather than local optimum. BayesOpt is a principled approach for approximating a probabilistic model of the objective function via a surrogate function to select future parameter values based on prior knowledge. In this study, BayesOpt is built on (1) a Tree-structured Parzen Estimator (TPE), a Bayesian surrogate model to fit results from objective function, and (2) an acquisition function to decide the next iteration of hyperparameter values.

In performing hyperparameter tuning, the dataset was initially split into 80% and 20% training and testing sets. The BayesOpt algorithm was given a search space of allowable values for all hyperparameters (see Supplementary Table S1), hence, providing a high likelihood of achieving a global optimum. The following is a five-step process for the hyperparameter selection:

1. For 50 independent trials, BayesOpt performed a parameter search on the respective baseline models, i.e., RF and SVM, using stratified 10-fold cross-validation on the training set while ensuring a minimized loss of the objective function.
2. After retrieving the set of hyperparameters that achieved a minimized loss, evaluate the performance of the optimized model by using the testing set.
3. Store the selected hyperparameters and their corresponding performances in a CSV file.
4. Repeat steps (1) to (3) for ten independent trials.
5. From the CSV file containing ten sets of hyperparameter values and their corresponding performance metrics, select the best set that achieved the highest performance accuracy. The optimal set of hyperparameters is demonstrated in Supplementary Table S2. Furthermore, to compare the model performances of using manual and automatic search algorithms, Supplementary Table S3 demonstrates that BayesOpt led to a significantly higher baseline performance.

#### Model classification

SVM is a well-known model classification algorithm that employs different kernel functions to map out input vectors from the low-dimensional space into a high-dimensional, hypothetical space. At its core, SVM constructs a hyperplane that adheres to the margin maximization principle, aiming to achieve the largest possible margin between the hyperplane and the nearest data points from each class. This principle ensures that the decision boundary is robust and generalizes well to new data, as it maximizes the separation between the classes and minimizes the risk of misclassification. Using the constructed hyperplane, the solution in distinguishing unseen samples with respect to the feature vector $x_i$ and a multiplier $\alpha _{i}$ that determines the orientation of the hyperplane is defined in Eq. (2).

2 $$\begin{aligned} f(\vec {x}) = sgn\,(\sum _{i=1}^{m} \, y_i \alpha _i \langle \vec {x}, \vec {x}_i \rangle + b) \end{aligned}$$

where *b* is the bias term that shifts the hyperplane away from the origin and the optimized sign function returns $+1$ if the feature vector lies on the positive class or $-1$ if the feature vector lies on the negative class. A detailed derivation of the SVM is discussed in Vossen et al.^41^.

The use of SVM was motivated by its ability to encapsulate two separate biological tasks in a unified manner: SNP-phenotype associations and phenotypic prediction. Rather than treating these tasks as separate entities, SVM applies a non-linear transformation on the SNP data. Using the constructed hyperplane, SVM then fits a non-linear model to distinguish points in the feature space. Such an approach simplifies the problem yet still accounting for the intricate interplay of disease-related biological features. Various literature^8,24,42–44^ similarly employed SVM to SNP data.

#### Model evaluation

All risk assessment models were evaluated using leave-one-out cross-validation (LOOCV) to ensure a stringent model evaluation. LOOCV is well-known for its stringent validation approach, where it iteratively leaves out one data point at a time as the validation set while using the remaining points for training. The division process is repeated until each observation has been used once as the testing data. In addition, model accuracy, sensitivity, precision, and area under the curve (AUC)-receiver operating characteristic (ROC), were determined for each model configuration for a more comprehensive model evaluation.

Within the LOOCV framework, feature selection was implemented to ensure generalizable feature importance scores. The feature selection process through RF involved a collective analysis of all 1,318,897 SNPs associated with the phenotype, rather than studying each SNP independently in isolation, to capture pertinent SNP-SNP interactions. Such a process involves iteratively retrieving SNP subsets ranging from 10 to 1000 SNPs (in intervals of 10), where each subset is subjected to RF training using LOOCV. The specified range of SNP subsets allows a balance between model complexity and generalization performance. Given N folds in LOOCV (where N = 200), the computation of the final feature importance per SNP subset is calculated as the average of all importance scores across N folds. Obtaining each feature subset involves selecting SNPs based on their computed highest feature importance scores. Specifically, a feature subset containing 10 SNPs was chosen based on the top 10 highest feature importance scores, and so on for higher-ranked subsets.

### Ethical approval

All participants provided written consents to take part in the study, permitting the use of their medical data and the collection of serum samples for research purposes. The project of Kim et al.^14^ received approval from the ethics committee at the Korea University Anam Hospital and was conducted in accordance with the ethical guidelines in the Declaration of Helsinki.

## Results

This study focuses on three main tasks: (1) assessing the predictive power of GWAS-identified candidate biomarkers, (2) assessing the predictive power of ML-identified candidate biomarkers, and (3) augmenting ML-identified candidate biomarkers to the core GWAS-based biomarkers model. It is worth noting that while the sample size used in this study may not be large enough, the balanced data distribution still enables a reliable evaluation of the models’ generalization abilities, as each class is fairly represented during each validation step in LOOCV. The use of LOOCV is deemed appropriate for small sample sizes as it maximizes the use of available data for training and validation, mitigating overfitting risk and minimizing the impact of chance associations within the data.

### Assessing the predictive power of GWAS-identified candidate biomarkers

To evaluate the predictive capacity of GWAS-identified candidate biomarkers, we constructed two risk assessment SVM models based on GWAS results: (i) the core GWAS-based biomarkers model that comprises the three most statistically associated GWAS-identified candidate biomarkers as indicated in Supplementary Table S4^14^ and (ii) the potential GWAS-based biomarkers model that includes 52 GWAS-identified candidate biomarkers with a cut-off *p*-value of $10^{-4}$ as indicated in Supplementary Table S5^14^. Conversely, a baseline model, which uses all SNPs from the data, was also developed to establish a benchmark. All risk assessment models were trained using the optimal set of hyperparameters of SVM from Supplementary Table S2.

Results from Table 1 indicate that selecting the most pertinent subset of features improves model performance since irrelevant features are removed from the data. Furthermore, the potential GWAS-based biomarkers model outperformed the core GWAS-based biomarkers model. Compared with the core GWAS-based biomarkers model comprising only three significantly relevant biomarkers, the potential GWAS-based biomarkers model demonstrated higher predictive capacity due to the more robust, collective signals provided by the 52 SNPs. From such an occurrence, the predictive power of the three GWAS-identified candidate biomarkers from Supplementary Table S4 is insufficient in developing a high-performing risk assessment model.

**Table 1:** Summary of risk assessment model performances.

Model	No. of SNPs	Accuracy	Precision	Sensitivity	Specificity	AUC
Baseline SVM model	1,318,897	0.64	0.71	0.39	0.86	0.64
Core GWAS-based biomarkers model	3	0.71	0.70	0.73	0.70	0.71
Potential GWAS-based biomarkers model	52	0.82	0.83	0.81	0.81	0.82
ML-based biomarkers model	150	0.80	0.79	0.80	0.79	0.80
GWAS+ML-based biomarkers model	960	0.90	0.89	0.90	0.89	0.90

### Assessing the predictive power of ML-identified candidate biomarkers

The ML-based biomarkers model, which uses ML-identified candidate biomarkers retrieved using the exhaustive feature selection via RF, was compared against the baseline SVM model (see Table 1). The implemented feature selection process aims to account for the non-random associations among SNPs and their joint effects, ensuring that this recognizes subtle yet robust relationships. Although some SNPs may individually have smaller effect sizes, they could be part of a larger gene network or genetic region with collective significance concerning the phenotype. With this, RF allowed capturing not only the SNPs with large effect sizes but also those with smaller effect sizes that might still be relevant in the context of linkage disequilibrium.

**Figure 2:**
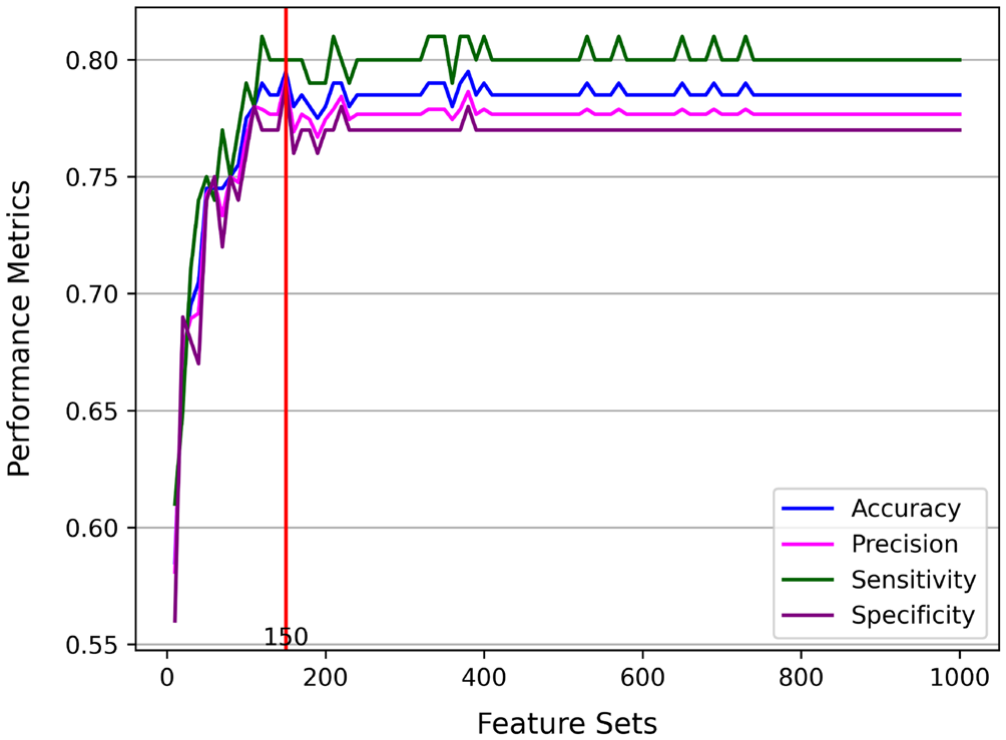
Performance metrics of ML-based biomarkers model with increasing feature set size.

The results showed that training the model using SNPs with high predictive power considerably improved model performance. As depicted in Fig. 2, the classification performances of the ML-based biomarkers model approach a converging value for an increasing number of features. Furthermore, as presented in Table 1 and Fig. 2, the ML-based biomarkers model attained maximum performance at a feature set of 150 SNPs, where Supplementary Table S6 demonstrates the highly-predictive SNPs identified by RF. Similarly, this further indicates that reducing the feature space enhances model performance by using collective interactions from multiple relevant variants.

From the feature selection process using RF, the calculated feature importance scores for the GWAS-identified candidate biomarkers from Supplementary Table S4 are 0.000041, 0.0000279, and 0.0000214 for rs6462008, rs171941, and rs7944135, respectively, with rs6462008 belonging to the feature set of 150 SNPs from Supplementary Table S6. From the feature set of 150, Table 2 shows the biological function of the top 5 SNPs with the highest predictive power. While GWAS has not identified the five ML-identified candidate biomarkers from Table 2 to have a high association (*p*-value $$< 10^{-4} $$) with HBsAg seroclearance, further investigations into their identifiable functional significance have revealed compelling results. Studies have established that the flanking genes linked to several ML-identified candidate biomarkers are linked to hepatocellular carcinoma (HCC). Cadherin 4 (CDH4), i.e., a gene linked to rs28588178, which attained the highest RF feature importance score, was established to have an association with the following diseases: HCC and craniofacial-deafness-hand syndrome. On the other hand, expression of tumor protein p53 inducible protein 11 (TP53I11), known as PIG11, was detected in HCC and normal liver tissues with an immohistochemical method^45^. Finally, PCED1B was reported to be upregulated with HCC with predicted poor survival^46^. Notably, the ML-based approach could investigate the biological link between HCC and HBsAg seroclearance. The result of the biomarker identification reinforces previous studies^47–53^ that clinical complications such as HCC are still possible even in the absence of HBsAg. As a result, this necessitates clinical monitoring and regular surveillance^47,54^.

**Table 2:** Biological function of the top 5 ML-identified candidate biomarkers.

SNP ID	Chromosome location	Gene
rs28588178	chr20:61355100	CDH4 : Intron variant
rs78736861	chr11:131240301	N/A
rs1994209	chr11:44951531	TP53I11 : 2KB upstream variant
rs2558276	chrY:6216488	N/A
rs7958186	chr12:47147485	PCED1B : Intron variant

### Augmenting ML-identified candidate biomarkers to the core GWAS-based biomarkers model

**Figure 3:**
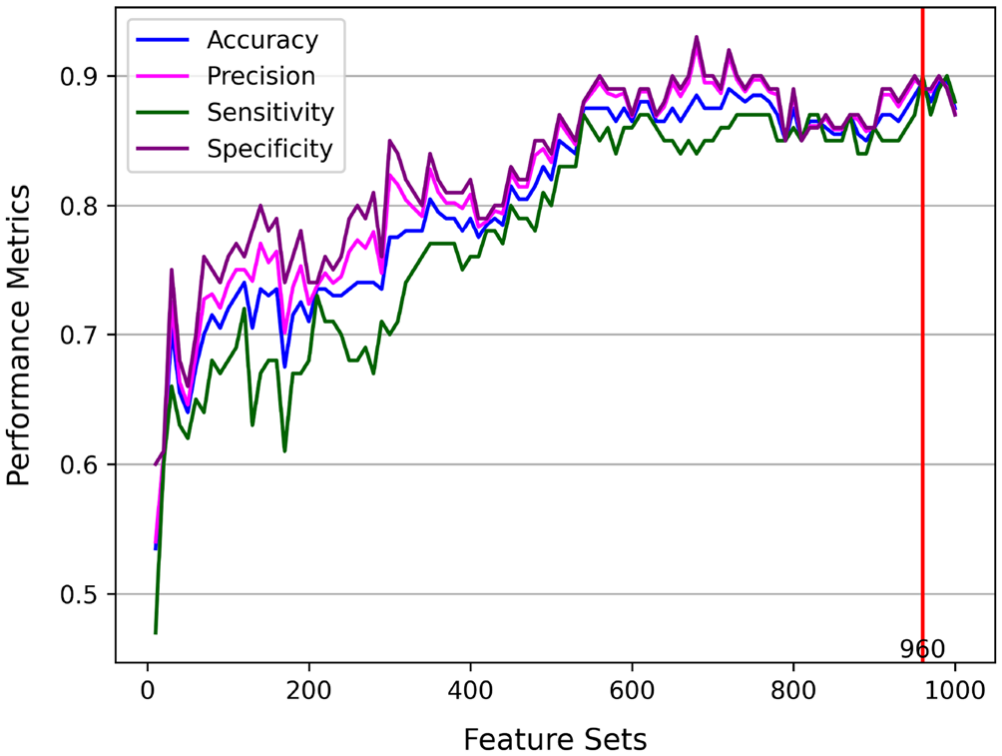
Model performance of GWAS+ML-based biomarkers model with increasing feature set size.

Finally, combining information from population and individual levels, we augmented the core GWAS-based biomarkers model by iteratively adding ML-identified candidate biomarkers retrieved through RF, referred to as the GWAS+ML-based biomarkers model. The mentioned model was developed to quantify the effect of the non-GWAS SNPs on the model’s predictive performance. As seen in Fig. 3, the added ML-identified candidate biomarkers to the GWAS-identified candidate biomarkers from Supplementary Table S4 resulted in a substantial increase in the model performance with maximum accuracy at a feature set of 960 SNPs, which surpassed the predictive performance of the previous risk assessment models as indicated in Table 1.

## Discussion

Unlike association studies, which are explanatory and focus on identifying patterns and relationships between variables based on the population-level, the prediction route in prediction studies such as ML is focused on developing models using the best combination of features with a final aim of personalized risk assessmentt^23^. To harness the complementary insights from population- and individual-levels, we assessed the predictive power of GWAS-identified candidate biomarkers, evaluated the predictive power of ML-identified candidate biomarkers, and developed a model based on the combination of SNPs identified using the two types.

Complex disorders are influenced by the joint contributions of multiple dysfunctional genetic variants, each of which contributes to the phenotypic expression with an individual effect of varying magnitude^20,55^. While utilizing various biomarkers causes a significant impact on the phenotype, the relevance of features must still be accounted for. For instance, signals originating from the three biomarkers from Supplementary Table S4 did not capture the pertinent biological interactions contributing to the phenotype due to the stringent thresholding employed in GWAS, leading to poor prediction performance. Such occurrence is consistent with the claim of Kooperberg et al.^56^, stating that utilizing multiple correlated SNPs rather than solely using the most statistically significant risk variants improves risk assessment model performance. More so, using the entire feature space without considering the relevance of the individual features hinders achieving an optimal model performance. Given that genomic datasets suffer from the curse of dimensionality^6,8,29–32^, it is crucial to eliminate irrelevant features and retain only the most informative variants related to the phenotype under investigation. Removing noise from the data improves models’ accuracy and reliability, thereby gaining a deeper understanding of the genetic mechanisms underlying risk susceptibility.

While identifying relevant genetic risk factors is a crucial step in assessing risk, a statistically significant association alone is inadequate to signify a claim of prediction^23^. Although association statistics provide valuable insights regarding the relationship between two variables, it does not guarantee a predictive relationship. For instance, the collective effects of the three GWAS-identified candidate biomarkers with robust associations from Supplementary Table S4 resulted in poor overall predictive validity, as demonstrated in the performance of the core GWAS-based biomarkers model. However, joint contributions of the 52 GWAS-identified candidate biomarkers with strong statistical associations (cutoff p-value $< 10^{-4}$) from Supplementary Table S5 exhibited strong claims of predictive utility, as seen in the performance of the potential GWAS-based biomarkers model. On another note, despite demonstrating exceptional predictive abilities of novel ML-identified candidate biomarkers in Supplementary Table S6, these lacked significant associations as they were not included among the GWAS-identified candidate biomarkers listed in Table S5. Overall, it is essential to recognize that information from association studies does not necessarily lead to accurate predictions, and insights from prediction studies do not essentially mean robust associations.

Understanding that association and prediction studies are not mutually exclusive, we used these approaches in conjunction with one another by combining information from the population and the individual levels. The superior model performance from the GWAS+ML-based biomarkers model illustrates that ML-based approaches could be employed as another approach in detecting collective effects of variants on complex traits^57^, thus aiding GWAS in identifying novel biomarkers. From these, incorporating the ML-identified candidate biomarkers into the three GWAS-identified candidate biomarkers from Supplementary Table S4 online suggests that ML is useful in the post-GWAS analysis^57^. Ultimately, the high performance attained by the GWAS+ML-based biomarkers model highlights the synergy of GWAS and ML in translating scientific discoveries into clinical and practical use.

## Conclusion

Acknowledging that association and prediction studies may offer complementary insights into disease mechanisms, we leveraged information at the individual and population levels to improve model performance. Iteratively adding ML-identified candidate biomarkers into the pool of the three most statistically significant GWAS-identified candidate biomarkers resulted in a considerable improvement in the model performance, attaining a maximum accuracy, sensitivity, precision, and AUC of 90%, 90%, 89%, and 0.90, respectively.

Extensive validation of all findings in the study is imperative in developing a robust and reliable personalized disease risk assessment through ML. To ensure the reliability of the proposed method, we recommend conducting validation studies that assess the utility of combining information from population and individual levels across various disease types and populations. Findings from this study are specific to the Korean cohort, and therefore, their generalizability necessitates further investigation. Verifying the biological mechanisms underlying GWAS-identified candidate biomarkers and ML-identified candidate biomarkers is also recommended to ensure that the identified biomarkers accurately indicate the phenotype. Without a clear understanding of the fundamental biology, biomarkers indicative of a phenotype may be inaccurate and unreliable, and their subsequent use in prognosis and diagnosis could be misguided.

### Supplementary Information

Supplementary Tables.

## Data Availability

The dataset used in this study is accessible through the figshare link: https://figshare.com/articles/dataset/gtReport_txt/6614975.
