# Supplementary material for: Genetic risk assessment based on association and prediction studies
Source: Sci Rep. 2023 Sep 14;13:15230. doi: 10.1038/s41598-023-41862-3 (PMC10502006; doi:10.1038/s41598-023-41862-3)
Supplement: Supplementary file 1 — Supplementary Tables. [file 41598_2023_41862_MOESM1_ESM.pdf]

Supplementary Information

Table S1. List of allowable hyperparameter values for each baseline model

Machine Learning Method	Hyperparameter	Brief Description	Search Space
RF	n_estimators	represents the optimum number of decision trees before taking the maximum averages of predictions	[1, 100]
	max_depth	portrays the maximum number of levels in each decision tree	[5, 50]
	min_samples_split	signifies the minimum number of points placed in a node before the node is split	[2, 11]
	min_samples_leaf	represents the minimum number of points in a leaf node	[1, 11]
	criterion	measures the quality of a split	{‘gini’}
	max_features	represents the maximum number of features for splitting a node	[1, 64]
SVM	C	represents the regularization parameter which represents misclassification or error term	[0.1, 50]
	kernel	accepts low dimensional input space and consequently transforms it into a higher-dimensional space	{'linear', 'poly', 'rbf'}

Table S2. Set of optimal hyperparameter values

Baseline Model	Hyperparameter	Optimal value
RF	n_estimators	42
	max_depth	6
	min_samples_split	10
	min_samples_leaf	10
	criterion	gini
	max_features	59
SVM	C, regularization parameter	0.97
	kernel	rbf

Table S3. Comparison of model performance achieved when using the set of hyperparameter values identified by BayesOpt and Manual Search

Hyperparameter Tuning Scheme	Baseline Model	Accuracy	Sensitivity	Precision
BayesOpt	RF	0.69	0.67	0.75
	SVM	0.64	0.37	0.71
Manual Search	RF	0.54	0.49	0.54
	SVM	0.50	0.49	0.50

Table S4. Core biomarkers identified by [2018 Kim]

rsID	Chr	Position	Cytoband	Gene	A1/A2	MAF		OR (95% CI)	P-value
						Case	Control		
rs6462008	7	27349479	7p15.2	EVX1, HOXA13	G/T	0.37	0.58	0.34(0.22-0.54)	3.40x10-6
rs171941	5	79180126	5q14.1	CMYA5, MTX3	A/G	0.38	0.19	3.69(2.13-6.42)	3.52x10-6
rs7944135	11	59020987	11q12.1	DTX4, MPEG1	A/G	0.31	0.13	4.16(2.27-7.63)	4.17x10-6

Table S5. Potential biomarkers identified by [2018 Kim]

rsID	Chr	Position	Cytoband	Gene	A1/A2	MAF		OR (95% CI)	P-value
						Case	Control		
rs6462008	7	27349479	7p15.2	EVX1, HOXA13	G/T	0.37	0.58	0.34(0.22-0.54)	3.40x10-6
rs171941	5	79180126	5q14.1	CMYA5, MTX3	A/G	0.38	0.19	3.69(2.13-6.42)	3.52x10-6
rs7944135	11	59020987	11q12.1	DTX4, MPEG1	A/G	0.31	0.13	4.16(2.27-7.63)	4.17x10-6
rs6462003	7	27336745	7p15.2	HOXA13	A/G	0.35	0.57	0.35(0.22-0.55)	7.26x10-6
rs199869387	2	59708649	2p16.1	-	A/G	0.56	0.35	2.97(1.84-4.79)	7.89x10-6
rs12463513	2	59644842	2p16.1	-	A/G	0.57	0.34	2.67(1.73-4.11)	8.85x10-6
rs6947275	7	27344799	7p15.2	HOTTIP, EVX1	C/G	0.36	0.57	0.35(0.22-0.56)	9.27x10-6
rs7230406	18	61863854	18q22.1	LOC284294	A/G	0.11	0.30	0.27(0.15-0.48)	1.09x10-5
rs13018470	2	59694749	2p16.1	-	T/C	0.56	0.36	2.92(1.81-4.72)	1.21x10-5
rs7945342	11	5543705	11p15.4	OR51B5	T/C	0.29	0.52	0.34(0.21-0.55)	1.26x10-5
rs2192611	2	59695598	2p16.1	-	A/C	0.56	0.35	2.91(1.80-4.70)	1.28x10-5
rs1809862	11	5537780	11p15.4	UBQLNL	A/C	0.29	0.51	0.34(0.21-0.55)	1.36x10-5
rs4377248	18	61862921	18q22.1	LOC284294	A/C	0.11	0.29	0.28(0.15-0.50)	1.92x10-5
rs62439524	7	2684070	7p22.3	TTYH3	G/C	0.57	0.39	2.94(1.79-4.81)	1.95x10-5
rs265005	5	79172136	5q14.1	CMYA5	C/T	0.33	0.16	3.24(1.87-5.60)	2.57x10-5
rs12464531	2	59673530	2p16.1	-	T/C	0.56	0.37	2.76(1.72-4.44)	2.82x10-5
rs2215905	2	59678265	2p16.1	-	C/T	0.56	0.37	2.76(1.72-4.44)	2.82x10-5
rs11891860	2	59667692	2p16.1	-	A/G	0.34	0.55	0.40(0.26-0.61)	3.20x10-5
rs1558599	2	59663978	2p16.1	-	T/C	0.34	0.56	0.40(0.26-0.62)	3.27x10-5
rs887941	2	59686958	2p16.1	-	C/T	0.56	0.37	2.69(1.69-4.30)	3.34x10-5
rs2017434	11	5536852	11p15.4	UBQLNL	G/A	0.32	0.53	0.37(0.23-0.59)	3.38x10-5
rs872751	11	5537045	11p15.4	UBQLNL	G/T	0.32	0.53	0.37(0.23-0.59)	3.38x10-5
rs2047456	11	5537161	11p15.4	UBQLNL	C/T	0.32	0.53	0.37(0.23-0.59)	3.38x10-5
rs12593003	15	69896675	15q23	DRAIC	A/G	0.15	0.35	0.32(0.18-0.55)	4.07x10-5
rs10769023	11	5536415	11p15.4	UBQLNL	T/C	0.32	0.53	0.37(0.23-0.60)	4.44x10-5
rs17584600	2	59661521	2p16.1	-	A/G	0.35	0.57	0.41(0.26-0.63)	4.49x10-5
rs8037510	15	86797384	15q25.3	AGBL1	A/G	0.15	0.32	0.32(0.18-0.55)	4.49x10-5
rs2153442	10	13511130	10p13	BEND7	G/A	0.32	0.13	3.15(1.81-5.47)	4.79x10-5
rs4748035	10	13511986	10p13	BEND7	T/C	0.32	0.13	3.15(1.81-5.47)	4.79x10-5
rs12620748	2	6866987	2p25.2	LINC01246	T/C	0.35	0.19	2.93(1.74-4.94)	5.18x10-5
rs6737829	2	59669528	2p16.1	-	C/T	0.39	0.22	2.79(1.70-4.58)	5.34x10-5

rs13033709	2	49490690	2p16.3	-	A/G	0.07	0.22	0.24(0.12-0.48)	5.92x10-5
rs12051751	17	16501887	17p11.2	CCDC144A, ZNF287	A/C	0.58	0.37	2.44(1.58-3.77)	6.31x10-5
rs2173091	15	86797399	15q25.3	AGBL1	G/A	0.17	0.35	0.35(0.21-0.58)	6.34x10-5
rs1546196	12	25085390	12p12.1	BCAT1	T/C	0.44	0.25	2.66(0.25-1.64)	6.60x10-5
rs1840440	18	23257186	18q11.2	-	C/T	0.37	0.57	0.37(0.23-0.60)	6.86x10-5
rs6840435	4	5813695	4p16.2	EVC	C/T	0.30	0.46	0.38(0.23-0.61)	7.07x10-5
rs3944255	11	59030285	11q12.1	DTX4, MPEG1	T/C	0.29	0.14	3.16(1.79-5.58)	7.11x10-5
rs9296504	6	46629505	6p12.3	SLC25A27	C/T	0.38	0.57	0.42(0.28-0.65)	7.44x10-5
rs9381469	6	46630068	6p12.3	SLC25A27	A/G	0.38	0.57	0.42(0.28-0.65)	7.44x10-5
rs9911677	17	16537397	17p11.2	ZNF624	T/C	0.59	0.38	2.42(1.56-3.75)	7.44x10-5
rs12151705	2	59738664	2p16.1	-	G/T	0.39	0.22	2.72(1.66-4.47)	7.46x10-5
rs7644288	3	172328281	3q26.31	-	A/T	0.20	0.07	4.06(2.03-8.16)	7.96x10-5
rs10508462	10	13519743	10p13	BEND7	G/T	0.32	0.14	2.99(1.73-5.16)	8.36x10-5
rs2191314	7	20355658	7p21.1	MACC1	T/C	0.48	0.30	2.61(1.62-4.22)	8.70x10-5
rs13382813	2	6852941	2p25.2	LINC01246	A/G	0.32	0.17	2.95(1.72-5.06)	8.76x10-5
rs10838245	11	5535529	11p15.4	OR51B5	T/G	0.32	0.52	0.38(0.24-0.62)	8.92x10-5
rs1482088	4	86559173	4q21.23	ARHGAP24	C/A	0.57	0.39	2.44(1.56-3.82)	9.18x10-5
rs7073919	10	98926961	10q24.1	SLIT1	G/A	0.10	0.24	0.28(0.14-0.53)	9.30x10-5
rs6749972\	2	59651584	2p16.1	-	T/C	0.36	0.56	0.42(0.28-0.65)	9.56x10-5
rs11931577	4	78335569	4q21.1	CXCL13	C/T	0.10	0.23	0.27(0.14-0.52)	9.85x10-5
rs6790457	3	156374086	3q25.31	LINC00886	A/G	0.21	0.08	3.96(1.98-7.92)	9.94x10-5

Table S6. Feature importance scores of the optimal 150 ML-identified candidate biomarkers

SNP	Feature Importance Score
rs28588178	7.00738E-05
rs78736861	6.35937E-05
rs1994209	6.23189E-05
rs2558276	5.96259E-05
rs7958186	5.92947E-05
kgp4951732	5.88591E-05
kgp2178698	5.83591E-05
kgp1641663	5.82342E-05
kgp2952352	5.56227E-05
kgp22820210	5.42226E-05
kgp1304141	5.40191E-05
kgp3310224	5.33998E-05
rs6697603	5.31115E-05
rs1704740	5.19973E-05
kgp9017174	5.14462E-05
rs2573958	5.11598E-05
rs7208308	5.07608E-05
rs2011198	5.04902E-05
kgp6249429	4.95222E-05
rs9586800	4.86620E-05
kgp11988514	4.85840E-05
kgp9168037	4.83831E-05
kgp529264	4.83179E-05
kgp22836517	4.83038E-05
rs818734	4.80730E-05
rs7287478	4.80687E-05
kgp5444803	4.80225E-05
rs10946795	4.79930E-05
rs10739387	4.78024E-05
rs9525826	4.77761E-05
rs4825302	4.74592E-05
rs7772549	4.72579E-05
kgp2275880	4.70879E-05
rs4726860	4.69771E-05
kgp11592074	4.69300E-05
kgp4049657	4.68916E-05

kgp6140766	4.68474E-05
kgp11841466	4.66663E-05
rs638130	4.66511E-05
kgp8418739	4.66444E-05
rs11936885	4.66352E-05
kgp22831089	4.65934E-05
rs2073359	4.64842E-05
kgp1173878	4.64841E-05
rs6064387	4.62657E-05
rs6888630	4.60082E-05
rs1391615	4.59790E-05
rs152523	4.59424E-05
kgp8918054	4.59178E-05
rs3948623	4.58502E-05
kgp8392062	4.56987E-05
kgp8065196	4.55987E-05
kgp888430	4.55473E-05
kgp12443907	4.54403E-05
rs7907842	4.54030E-05
kgp22745700	4.53212E-05
kgp5643734	4.52939E-05
kgp5617407	4.52286E-05
kgp6112872	4.51138E-05
kgp933515	4.50045E-05
kgp8606888	4.49812E-05
kgp6527432	4.48255E-05
kgp3464797	4.47484E-05
kgp5344688	4.45061E-05
rs425181	4.43235E-05
rs12345947	4.42977E-05
rs12635437	4.41717E-05
kgp3652622	4.41183E-05
rs12663566	4.38410E-05
rs4909820	4.37506E-05
rs744892	4.37099E-05
rs2403238	4.35838E-05
rs13304828	4.35460E-05
kgp12202614	4.33603E-05
rs1485096	4.32918E-05
kgp22795303	4.32908E-05

kgp10343661	4.32648E-05
rs2114601	4.32260E-05
kgp4108382	4.31520E-05
kgp1650935	4.31139E-05
kgp11433992	4.30459E-05
kgp9068128	4.29869E-05
rs206906	4.29693E-05
rs2345237	4.29655E-05
kgp11517345	4.29630E-05
kgp11860699	4.29460E-05
kgp22730965	4.29441E-05
kgp10565089	4.28529E-05
kgp8750477	4.28046E-05
kgp22789258	4.27728E-05
rs4377248	4.27546E-05
kgp2437592	4.26211E-05
kgp10581464	4.26052E-05
rs4854951	4.25990E-05
kgp7852726	4.25889E-05
kgp1574687	4.24944E-05
kgp2484003	4.24718E-05
kgp7591659	4.23225E-05
kgp103376	4.23218E-05
kgp6875244	4.23131E-05
kgp5747010	4.22863E-05
rs2685891	4.22766E-05
kgp1403654	4.22224E-05
kgp8352395	4.22063E-05
kgp22823137	4.21036E-05
kgp5889025	4.20949E-05
kgp7474144	4.20466E-05
kgp5274176	4.20307E-05
rs11181991	4.20122E-05
kgp969028	4.19687E-05
rs1827101	4.19304E-05
kgp4855340	4.18524E-05
rs10803930	4.18331E-05
kgp728097	4.18310E-05
kgp9636562	4.18107E-05
kgp9068539	4.18027E-05

kgp22748307	4.18025E-05
kgp4380013	4.17173E-05
kgp1467847	4.16715E-05
rs1403501	4.16612E-05
rs4406360	4.15893E-05
kgp1964199	4.15836E-05
kgp11289793	4.15582E-05
rs10866820	4.14535E-05
kgp22798606	4.13821E-05
rs934465	4.13612E-05
kgp1971589	4.13233E-05
rs1546658	4.11543E-05
rs2243626	4.11095E-05
rs6971826	4.10952E-05
rs3748269	4.10346E-05
rs243889	4.10235E-05
kgp2625682	4.10027E-05
rs5963772	4.09249E-05
kgp21283251	4.09119E-05
kgp12477583	4.09099E-05
kgp3378427	4.08498E-05
kgp10992035	4.08461E-05
kgp6113667	4.08277E-05
rs6462008	4.08262E-05
kgp8664316	4.07841E-05
rs12412964	4.07802E-05
rs815863	4.07741E-05
rs10877071	4.06796E-05
rs4515818	4.06385E-05
rs749622	4.06198E-05
kgp9803515	4.05942E-05
kgp7664778	4.05811E-05
rs2557743	4.05529E-05
rs9569064	4.05401E-05
